# Analysis of nuclear maturation, DNA damage and repair gene expression of bovine oocyte and cumulus cells submitted to ionizing radiation

**DOI:** 10.1590/1984-3143-AR2023-0021

**Published:** 2023-05-29

**Authors:** Bruno Tomazele Rovani, Vitor Braga Rissi, Monique Tomazele Rovani, Bernardo Garziera Gasperin, Tadeu Baumhardt, Vilceu Bordignon, Liliane de Freitas Bauermann, Daniele Missio, Paulo Bayard Dias Gonçalves

**Affiliations:** 1 Laboratório de Biotecnologia e Reprodução Animal, Universidade Federal de Santa Maria, Santa Maria, RS, Brasil; 2 Laboratório de Reprodução Animal, Grupo FiBRA, Universidade Federal de Pelotas, Pelotas, RS, Brasil; 3 Serviço de Radioterapia, Hospital Universitário de Santa Maria, Universidade Federal de Santa Maria, Santa Maria, RS, Brasil; 4 Department of Animal Science, McGill University, Sainte Anne de Bellevue, QC, Canada; 5 Departamento de Fisiologia e Farmacologia, Universidade Federal de Santa Maria, Santa Maria, RS, Brasil

**Keywords:** ionizing radiation, infertility, oocyte, DNA damage

## Abstract

Radiotherapy causes destruction of tumor cells, but also threatens the integrity and survival of surrounding normal cells. Then, woman submitted to irradiation for cancer treatment may present permanent ovary damage, resulting in impaired fertility. The objective of this study was to investigate the effects of therapeutic doses of ionizing radiation (IR), used for ovarian cancer treatment in humans, on bovine cumulus-oocyte complexes (COCs) as experimental model. Bovine ovaries were exposed to 0.9 Gy, 1.8 Gy, 3.6 Gy or 18.6 Gy IR, and then COCs were collected and used to evaluate: (a) oocyte nuclear maturation; (b) presence of phosphorylated H2A.X (γH2AX), as an indicator of DNA double-strand breaks (DSBs); and (c) expression of genes involved in DNA repair (*TP53BP1, RAD52, ATM, XRCC6* and *XRCC5*) and apoptosis (*BAX*). The radiation doses tested in this study had no detrimental effects on nuclear maturation and did not increase γH2AX in the oocytes. However, IR treatment altered the mRNA abundance of *RAD52* (RAD52 homolog, DNA repair protein) and *BAX* (BCL2-associated X protein). We conclude that although IR doses had no apparent effect on oocyte nuclear maturation and DNA damage, molecular pathways involved in DNA repair and apoptosis were affected by IR exposure in cumulus cells.

## Introduction

The medical application of ionizing radiation (IR) has been crucial in several cancer treatment modalities due to its ability to destroy the carcinogenic cells. It is estimated that more than 50% of diagnosed cancer patients receive radiation therapy (alone or in combination with chemotherapy or surgery) (Atun et al., 2015; Ding et al., 2013). This treatment has been responsible for improvement in the survival rates, especially children and adolescents (Chemaitilly et al., 2006; Ward et al., 2014). The main target of radiotherapy is the Deoxyribonucleic acid (DNA) of the carcinogenic cells, which is very sensitive to deleterious effect of IR (Hosseinimehr, 2015), which can induce DNA damage directly or indirectly (Kocakavuk et al., 2021). The direct effect induces a one-electron oxidation of DNA, while the indirect effect generates reactive oxygen species (ROS) through water radiolysis, which can induce DNA damage (Mavragani et al., 2019; Nikitaki et al., 2015). The direct effect results in single-strand breaks (SSB) and/or double-strand breaks (DSB) that may follow in mutations and widespread structural rearrangement of the genome, potentially causing cell death by apoptosis (Lord and Ashworth, 2012; Mavragani et al., 2019). Radiotherapy causes destruction of tumor cells through these mechanisms, but also threatens the integrity and survival of surrounding normal cells (Ahmed et al., 2014; Kumari et al., 2020; Zhou, 2020). Thus, the damage to healthy cells may also occur in radiation therapy, and the ovary is a target organ for anti-proliferative treatments (Lo Presti et al., 2004).

Woman treated for cancer with abdominal, pelvic or total body irradiation may present permanent ovary damage and loss of primordial follicles, resulting in impaired fertility and a premature menopause (Marci et al., 2018; Wallace et al., 2005, 2003). Age at the time of exposure to radiotherapy, extent and type of radiation therapy (e.g., abdominal or pelvic external beam irradiation), and fractionation schedule are important prognostic indicators for ovarian failure establishment (Muñoz et al., 2016; Sonmezer and Oktay, 2004). It has been showed that, whereas an effective sterilizing dose at birth is 20.3 Grays (Gy = radiation absorbed dose unit), at 10 years old, it is 18.4 Gy; at 20 years old, 16.5 Gy; and at 30 years old, 14.3 Gy (Wallace et al., 2005). Through a mathematical model, it was demonstrated that less than 2 Gy is enough to destroy half of the human oocyte population (LD50) and more than 6 Gy usually causes irreversible ovarian failure (Wallace et al., 2003). Ovarian failure has been reported in 90% of patients followed up long term after total body irradiation (10-15 Gy, ~2 Gy per fraction), and in 97% of the females treated with fractionated total abdominal irradiation (20-30 Gy, 1-2 Gy per fraction) during childhood (Meirow et al., 2010; Wallace et al., 1989). Young patients treated with chemotherapy or radiotherapy, who can expect a normal life span, may suffer significant damage to the ovary (Hessels et al., 2022; Levine et al., 2018; Lo Presti et al., 2004). Consequently, early and late effects after treatment are gaining greater importance for survivors and their families (Tonorezos et al., 2022; Yurut-Caloglu et al., 2015).

In this context, further studies on the mechanisms involved in the interaction of ionizing radiation with the reproductive system become necessary to prevent or reduce potential damage and develop new alternatives for fertility preservation. Thus, studies on animal models have been performed to investigate the effects of IR on the female gamete (Pesty et al., 2010; Puy et al., 2021). Bovine females have been established as a model for the study of human ovarian folliculogenesis due to similarities in the dynamics of follicle development (Baerwald, 2009; Sirard, 2017), endocrine control (Malhi et al., 2005), single ovulation, as well as aged oocyte (Soares et al., 2020) and embryo metabolism (Langbeen et al., 2015). It is noteworthy that the search for models that closely reflect human biology is a major challenge, especially related to *in vitro* models.

Therefore, this study was designed to evaluate the effects of the standard (1.8 Gy), half (0.9 Gy) and double (3.6 Gy) fractional therapeutic doses of IR used for human ovarian cancer treatment on bovine cumulus-oocyte complexes matured *in vitro* on: (a) oocyte nuclear maturation; (b) presence of γH2AX in oocytes DNA; and (c) expression of genes involved in DNA repair (*TP53BP1, RAD52, ATM, XRCC6* and *XRCC5*) and apoptosis (*BAX*) in cumulus cells.

## Methods

All chemicals were purchased from Sigma Chemicals Company (St. Louis, MO, USA), unless otherwise indicated in the text. All procedures involving ovarian irradiation were approved by the HUSM Hospital Research Committee (#118/2013) from Federal University of Santa Maria.

### Collection and irradiation of the ovaries

**Figure 1:**
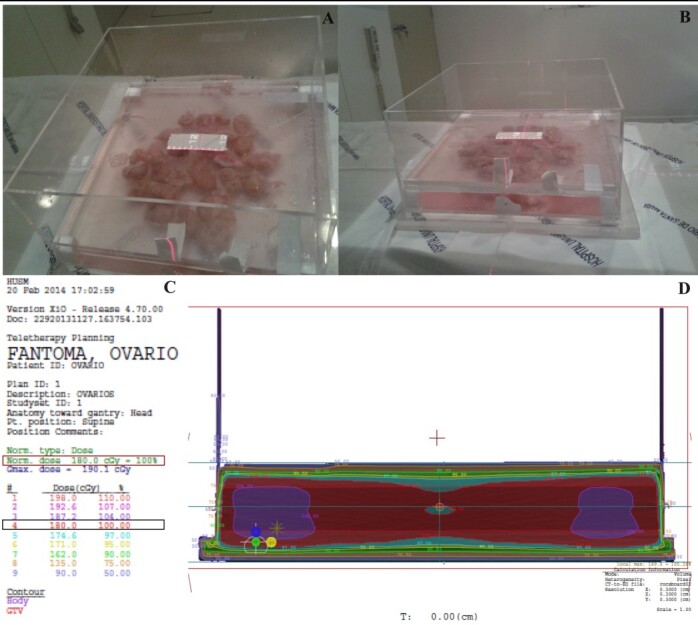
Procedure for ionizing radiation exposure of bovine ovaries. Representative image of bovine ovaries in the acrylic tank during irradiation procedure (A and B). Representative phantom obtained by computed tomography performed to validate the exposure to specific radiation doses, ensuring that all the ovaries received the desired radiation dose in the center of the acrylic tank (C and D). The desired dose of radiation is observed in the highlighted red box (in that case 180.0 cGy = 1.8 Gy) and 100% of the desired radiation was obtained in the center of the acrylic tank (D) highlighted in the black box demonstrated (C).

Cow ovaries were obtained from a local abattoir, stored on isothermal vials with saline solution (0.9% NaCl at 30°C) containing penicillin (100 IU/mL) and streptomycin (50 μg/mL) and transported to the Radiotherapy Service of Santa Maria University Hospital (Santa Maria, RS, Brazil). A portion of the ovaries (n = 80; 20/group) was irradiated in an acrylic tank (30 cm x 30 cm x 15 cm) containing saline solution with an Elekta linear accelerator, Precise System model (Elekta, Stockholm, Sweden). Another portion of the ovaries was not submitted to irradiation (control group; n = 20 ovaries). The experiments were conducted in three replicates, always with 20 ovaries per group in each replicate. Prior to irradiation, a Computed Tomography (CT) was performed in the acrylic tank to mimic the irradiation experiments and ensure that the ovaries received 100% of the radiation dose. Representative images of the ovaries in the acrylic tank containing saline and the validation of radiation dose are provided in Figure 1. The tested radiation doses correspond to standard daily fraction dose for ovarian cancer treatment (1.8 Gy), half and double of that (0.9 Gy and 3.6 Gy, respectively) and the equivalent dose of a total treatment adapted for a single session of irradiation (18.6 Gy), considered a sterilizing dose when used at puberty (Wallace et al., 2005). The irradiation procedures were previously authorized by the Hospital Research Management and conducted according to internal protocols.

### In vitro maturation (IVM)

After irradiation, the ovaries (n = 20 ovaries/group) were transported from the Hospital to the Laboratory of Biotechnology and Animal Reproduction in saline solution (0.9% NaCl, 30 °C). Cumulus-oocyte complexes (COC) were aspirated with a vacuum pump (suction flow rate of 15 mL/min) from 2 to 8 mm diameter follicles. Grade 1 and 2 COCs from each group were selected according to a previously published methodology (Leibfried and First, 1979) under a stereomicroscope and cultured in 400 μL maturation medium (Barreta et al., 2012). The COCs were cultured in an incubator at 39 ^o^C in a saturated humidity atmosphere containing 5% CO_2_ and 95% air for 24 h. The experiment was performed in three replicates.

### Assessment of nuclear maturation

After the *in vitro* maturation period, cumulus cells were removed by mechanical stirring. A total of 272 oocytes were evaluated (Control: n = 64; 0.9 Gy: n = 50; 1.8 Gy: n = 54; 3.6 Gy: n = 55 and 18 Gy: n = 49) in three replicates. The oocytes were fixed in 4% paraformaldehyde for 15 min at room temperature and transferred to a 0.5% Triton X-100 in PBS solution. For the evaluation of nuclear maturation, oocytes were exposed to 10 μg/ml bisbenzimide (Hoechst 33342) for 15 min. Stained oocytes were classified under UV light (wavelength of 340-380 nm) in a fluorescence microscope and considered mature if it displayed a chromatin configuration corresponding to metaphase II stage.

### Immunofluorescence detection of phosphorylated histone H2AX

A total of 172 oocytes were evaluated (Control: n = 62; 1.8 Gy: n = 51 and 18 Gy: n = 59) in three replicates. Oocytes were fixed for 15-20 minutes in 4% paraformaldehyde and permeabilized in 1% Triton X-100 in PBS (30 min at 37 ^o^C). Fixed oocytes were then washed twice in blocking solution (3% BSA and 0.2% Tween-20 in PBS) and maintained overnight in the presence of the anti-phospho-H2AX (Serine 139) mouse monoclonal primary antibody (Upstate Cell Signaling Solutions, NY, USA) diluted in blocking solution (1:1000). Oocytes were then washed thrice for 20 min each in blocking solution and incubated for 1 h at room temperature (24-26 °C) in the presence of 1:500 diluted anti-mouse IgG Alexa 394^®^ (Life Technologies, Burlington, ON, CAN). Samples were washed twice (20 min each) in blocking solution. After this period, DNA was stained by exposing the samples to 10 µg/mL of 4’, 6- diamidino-2-phenylindole (DAPI; Life technologies) in the blocking solution for 20 min. The samples were then washed (20 min) with the blocking solution and mounted on microscope slides using a drop of Mowiol. The slides were kept in a dark box and examined by epifluorescence using a Leica DMI4000B microscope (Leica Microsystems, Buffalo Grove, IL, USA). Images were recorded using a 340FX digital fluorescence camera and Leica LAS software (Leica Microsystems). Nuclei were evaluated for the immunostaining signal and classified as positive or negative.

### RNA extraction and qRT-PCR

Cumulus cells from 172 COCs were evaluated (Control: n = 62; 1.8 Gy: n = 51 and 18 Gy: n = 59) in three replicates. Cumulus cells were obtained after 1 h of IVM through hyaluronidase treatment (0.1% hyaluronidase in TCM199) and mechanical stirring for 5 min. Total RNA was extracted from cumulus cells using Trizol protocol (Life Technologies) according to the manufacturer’s instructions. Quantitation and estimation of RNA purity was performed using a NanoDrop (Thermo Scientific - Waltham, USA; Abs 260/280 nm ratio) spectrophotometer. Ratios above 1.8 were considered pure, and samples below this threshold were discarded. Complementary DNA was synthesized from 300 ng RNA, which was first treated with 0.1 U DNase, Amplification Grade (Life Technologies) for 5 min at 37 °C. After DNase inactivation at 65 °C for 10 min, the samples were incubated in a final volume of 20 µL with iScript cDNA Synthesis Kit (BioRad, Hercules, CA, USA). Complementary DNA synthesis was performed in three steps: 25 °C – 5 min, 42 °C – 30 min and 85 °C – 5 min. Quantitative polymerase chain reactions (qPCR) were conducted in a CFX384 thermocycler (BioRad) using SoFast EvaGreen supermix (BioRad) and bovine specific primers (Table 1) taken from the literature or designed using the Primer Express Software (Thermo Scientific). Standard two-step qPCR was performed with initial denaturation at 95ºC for 5 min followed by 40 cycles of denaturation at 95 ºC for 15 sec and annealing/extension at 58 ºC for 30 sec. Melting-curve analyses were performed to verify product identity. To optimize the qPCR assay, serial dilutions of cDNA templates were used to generate a standard curve. The standard curve was constructed by plotting the log of the starting quantity of the dilution factor against the Ct value obtained during the amplification of each dilution. Reactions with a coefficient of determination (R2) higher than 0.98 and efficiency between 95 to 105% were considered optimized. The relative standard curve method was used to assess the amount of a particular transcript in each sample. Samples were run in duplicate, and results are expressed in relation to the average Cq values for Cyclophilin B (*PPIB*) and Histone *H2A* as internal controls.

**Table 1:** List of primers used in the qPCR analyses. The primer sequences and concentrations used to amplify each gene are described.

**List of primers used in the qPCR analyses**			
***PPIB***	F: GGTCATCGGTCTCTTTGGAA	200	NM_174152.2
	R: TCCTTGATCACACGATGGAA	200	
***Histone H2A***	F: GAGGAGCTGAACAAGCTGTTG	200	(Bettegowda et al., 2006)
	R: TTGTGGTGGCTCTCAGTCTTC	200	
***TP53BP1***	F: ATCAGACCAACAGCAGAATTTCC	200	ENSBTAT00000028388
	R: CACCACGTCAAACACCCCTAA	200	
***RAD52***	F: GGCCAGGAAGGAGGCAGTA	200	ENSBTAT00000055617
	R: TGACCTCAGATAGTCTTTGTCCAGAA	200	
***ATM***	F: CTTAGGAGGAGCTTGGGCCT	200	ENSBTAT00000040104
	R: CCGCTGTGTGGCAAACC	200	
***BAX***	F: GACATTGGACTTCCTTCGAGA	200	ENSBTAT00000017739
	R: AGCACTCCAGCCACAAAGAT	200	
***XRCC6***	F: AATTGACTCCTTTTGACATGAGCAT	200	NM_001192246.1
	R: CCATAGAACACCACTGCCAAGA	200	
***XRCC5***	F: TGGCATCTCCCTGCAGTTCT	200	NM_001102141.1
	R: AGGCCCATGGTGGTCTGA	200	

F: forward primers; R: reverse primers.

### Experimental design

In Experiment 1, after irradiation of the ovaries (0 Gy (control), 0.9 Gy, 1.8 Gy, 3.6 Gy, and 18.6 Gy; n = 20 ovaries/group/replicate), grade 1 and 2 COC were cultured in 400 μL of maturation medium, as described above. After 24 h of MIV, oocyte was used for nuclear maturation assessment. The experiment was conducted in three replicates.

In Experiment 2, after irradiation of the ovaries (0 Gy (control), 1.8 Gy and 18.6 Gy; n = 20 ovaries/group/replicate), the same procedures of Experiment 1 were performed for IVM. However, after 1 h in IVM, cumulus cells were separated from the oocytes and used to quantify mRNA abundance of genes involved in: (a) DNA repair mechanisms including *TP53BP1, RAD52, ATM, XRCC6* and *XRCC5*; and (b) apoptosis process including the BCL2-associated X protein (*BAX*). Gene expression was assessed through quantitative Real-Time PCR techniques. At the same time, oocytes were selected to assess the presence of γH2AX foci, as indicator of DNA double-breaks. The experiment was conducted in three replicates.

### Statistical analysis

Maturation data were analyzed using the ANOVA test in a statistical model for categorical data, using the PROC CATMOD (Categorical Data Analysis Procedures) by SAS software (©SAS Institute, Inc., Cary, NC, USA). Variation in mRNA abundance was analyzed by ANOVA and multiple comparisons among groups were performed by LSMeans Student’s t test using the JMP Software (JMP® 7.0, SAS Institute, Inc., Cary, NC, USA). Continuous data were tested for normal distribution using Shapiro-Wilk test and normalized when necessary. In all analyses a P ≤ 0.05 was considered statistically significant. Non-categorical data are presented as means ± SEM.

## Results

### Nuclear maturation of oocytes

**Figure 2:**
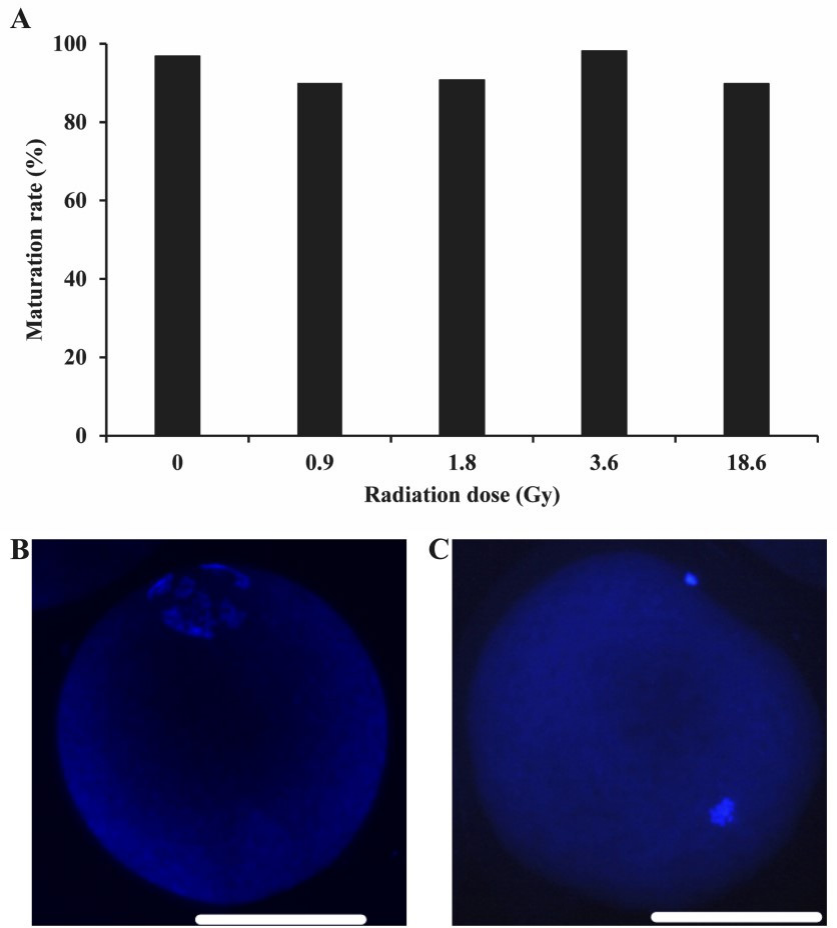
Effect of radiation dose (Gy) on nuclear maturation of bovine oocytes after 24 h in vitro culture. A total of 272 oocytes were evaluated (Control: n = 64; 0.9 Gy: n = 50; 1.8 Gy: n = 54; 3.6 Gy: n = 55 and 18 Gy: n = 49) in three replicates. Oocytes were considered mature if it displayed a chromatin configuration corresponding to metaphase II stage (A). Representative image of an immature (germinal vesical stage) (B) and a mature (metaphase II stage) (C) oocyte. White bars = 100 μm.

Nuclear maturation is considered a parameter of the viability and competence of the oocyte. In our study, there was no difference in nuclear maturation among groups, and high levels of maturation were identified in all IR tested doses (Figure 2). Despite the analysis having demonstrated no difference among groups, this does not mean that they did not suffer DNA damage. Then, we carried out immunofluorescence detection of phosphorylated histone H2AX to verify possible DNA damage.

### Immunofluorescence histone γH2AX detection

**Figure 3:**
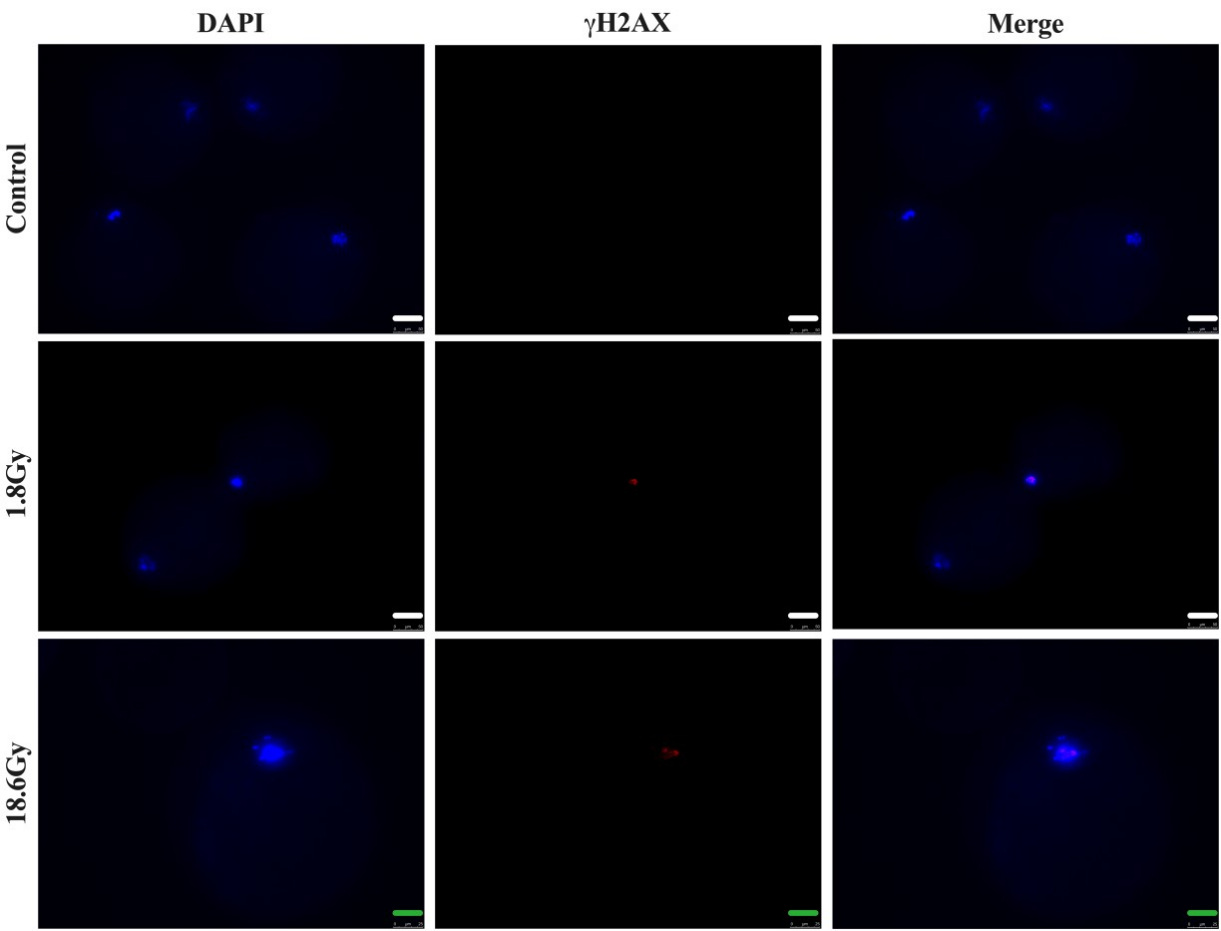
Immunodetection of phosphorylated histone H2AX 1 h after irradiation with a linear accelerator. Control (non-irradiated group; n = 20 oocytes), 1.8 Gy (n = 20 oocytes) and 18.6 Gy (n = 20 oocytes). A total of 172 oocytes were evaluated (Control: n = 62; 1.8 Gy: n = 51 and 18 Gy: n = 59) in three replicates. White bars = 50 μm; green bars = 25 μm.

One of the earliest responses to DSB induction is the phosphorylation of histone variant H2AX at the break sites. The γH2AX forms “foci” at the sites of DSB-induced by ionizing radiation, which can be detected by immunofluorescence with specific antibodies. In our study, we tested the radiation doses of 1.8 Gy and 18.6 Gy, in order to verify a possible DNA damage through immunodetection of histone γH2AX foci. We did not detect significant damage 1h after exposure to radiation doses. Immunofluorescence of γH2AX was only detected in two oocytes, one from the 1.8 Gy and one from the 18.6 Gy treatments, out of twenty oocytes analyzed per group (Figure 3).

### Assessment of gene expression

**Figure 4:**
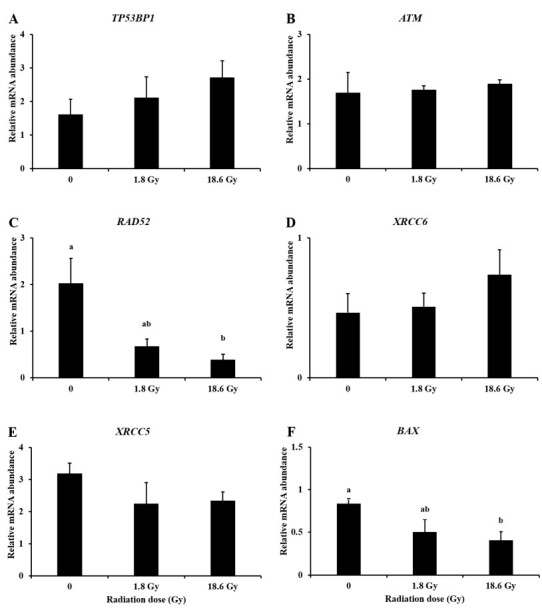
Relative mRNA abundance (mean ± standard error of mean) of *TP53BP1* (A), *ATM* (B), *RAD52* (C), *XRCC6* (D), *XRCC5* (E) and *BAX* (F) in bovine cumulus cells 1 h after irradiation with a linear accelerator. Different letters indicate statistical difference among groups (P < 0.05). Cumulus cells from a total of 172 COCs were evaluated (Control: n = 62; 1.8 Gy: n = 51 and 18 Gy: n = 59) in three replicates.

Since DSB are repaired through homologous recombination (HR) and non-homologous end joining (NHEJ) in mammalian cells, we attempted to analyze some genes involved in these pathways (*TP53BP1, RAD52, ATM, XRCC6* and *XRCC5*), as well as apoptosis (*BAX*). There was no significant variation in *TP53BP1* and *ATM* mRNA abundance (Figure 4A-4B). However, a slightly increase in *TP53BP1* mRNA was observed as the radiation dose increased (Figure 4A). On the other hand, the increasing radiation doses reduced the abundance of mRNA encoding the RAD52 protein (P < 0.05; Figure 4C). There was no effect of treatment on the mRNA abundance of the genes *XRCC6* and *XRCC5*, which are involved in the NHEJ pathway (Figure 4D-4E). The analysis of pro-apoptotic gene *BAX* showed a decrease of mRNA abundance when increasing the radiation dose (Figure 4F).

## Discussion

In this study, two experiments were conducted to investigate the biological consequences of ionizing radiation on bovine COCs obtained from antral follicles. The main findings of this study are: (1) the radiation doses tested (0.9 Gy, 1.8 Gy, 3.6 Gy and 18.6 Gy) did not affect the oocyte nuclear maturation rate; (2) DNA damage in oocytes was not increased by IR (1.8 Gy and 18.6 Gy), as assessed by γH2AX immunofluorescence 1h after treatment; (3) IR induced significant changes in mRNA abundance of *RAD52* and *BAX* in cumulus cells 1h after treatment.

Nuclear maturation refers to the progression of meiosis from the diplotene of prophase I to metaphase II, the stage when oocyte acquires the ability to regulate fertilization and support normal embryo development (Gottardi and Mingoti, 2010). Findings in this study revealed that maturation process was not affected by the IR doses tested. These data suggest that if there was irradiation-induced DNA damage, it was probably repaired, allowing the oocyte to develop to the metaphase II stage. In addition, we found that presence of fluorescent foci for γH2AX was not increased by IR treatment. One of the earliest responses to DNA damage is the phosphorylation of histone variant H2AX, which forms foci around the DSB sites (Firsanov et al., 2011; Rogakou et al., 1999, 1998). The γH2AX foci play an important role in identifying DNA damage and promoting repair by allowing sensor and repair proteins to access the damaged sites (Sudhakaran et al., 2015). In an experiment with human lymphocytes submitted to different radiation doses, a maximum response of γH2AX levels was observed at 1 or 2h, which was followed by a gradual loss of γH2AX over the next 6h, and a slower decline until 24h toward background levels (Ghardi et al., 2012). However, we did not detect significant γH2AX increase at 1h after IR exposure. This suggests that the IR tested dosed did not induce DNA damage in the oocytes or the phosphorylation of H2AX takes more to be completed in the oocyte than somatic cells (MacPhail et al., 2003). It is also possible that radiosensitivity differences between individuals and species may account for the results (Adam-Guillermin et al., 2018; Adriaens et al., 2009; Beaugelin-Seiller et al., 2020; Shim et al., 2016).

To gain additional insights on IR effects on COCs the mRNA abundance of genes involved in HR and NHEJ pathways for DNA repair, as well as apoptosis gene was evaluated in cumulus cells. DNA DSB are mainly repaired through the HR and the NHEJ repair pathways (Trenner and Sartori, 2019; Ruiz-Herrera et al., 2012). Mammalian oocytes express DNA repair genes and are capable of repairing DNA damage (De Felici and Klinger, 2011; Barreta et al., 2012; Martin et al., 2019; Ménézo et al., 2010; Winship et al., 2018). The mechanisms that detect and repair DSB, which are the most deleterious form of DNA damage, are especially relevant in radiobiology (Moscariello et al., 2015). Proteins involved in the HR pathway include RAD51, RAD52, ATM and TP53BP1, and those involved in the NHEJ pathway include the Ku70/Ku80 heterodimer (Jensen and Rothenberg, 2020; Ristic et al., 2003). The tumor protein p53 binding protein 1 (TP53BP1) is a nuclear protein that rapidly localizes the sites of DSB induced by ionizing radiation (peak detection between 30 min to 1 h) (Lei et al., 2022; Rappold et al., 2001; Schultz et al., 2000; Ward et al., 2003), participates in DNA damage signaling pathways and is regulated by ATM after IR (Rappold et al., 2001). Once activated, ATM phosphorylates numerous substrates in the cell and induces the cell response to DNA damage via facilitating DNA repair and modulation of cell cycle arrest (Gatei et al., 2003; Shiloh and Ziv, 2013; Weber and Ryan, 2015). In our analysis, there was no significant variation in *TP53BP1* and *ATM* mRNA abundance on the tested doses 1 h after irradiation. However, an increase in *TP53BP1* expression was observed as the radiation dose increases, suggesting an increase in DNA damage in cumulus cells.

Another protein involved in homologous recombination is the RAD52, that acts at the earliest stage of homologous DNA repair, playing a key part in the recognition and binding of double-strand breaks (Hiom, 1999). Besides, RAD52 binds selectively to DNA ends, protects these ends from digestion by exonucleases and promotes DNA end joining (Hiom, 1999). Repair of double-strand breaks is critical for maintenance of genomic integrity and cell survival. We observed in this study that *RAD52* mRNA decreased in cumulus cells exposed to increasing IR doses, indicating a possible reduction of cellular viability (Park, 1995). In relation to the NHEJ pathway, we evaluated the mRNA abundance of *XRCC6* and *XRCC5* genes (encoding for Ku70 and Ku80 protein, respectively). Ku is a heterodimer of two proteins, Ku70 and Ku80, and plays a critical part in NHEJ. Mammalian cells that lack either Ku70 or Ku80 are deficient in NHEJ repair and exhibit extreme sensitivity to radiation (Hiom, 1999; Vandersickel et al., 2010). In our analysis, there was no significant variation in *XRCC6* and *XRCC5* mRNA abundance. However, it was observed an increase in *XRCC6* and a decrease in *XRCC5* expression when increasing the radiation dose. These data raise the hypothesis that the irradiation rise in *XRCC6* mRNA abundance may indicate a rapid transcription and the decrease in *XRCC5* mRNA abundance may indicate a rapid translation. It is known that eukaryotic cells can activate an apoptotic mechanism in response to IR (Gong et al., 1999; Panganiban et al., 2013). BAX is a member of the Bcl-2 family of proteins and functions as a pro-apoptotic death effector (Chong et al., 2000). Because the cell cycle is affected by irradiation, and radiosensitivity depends on cell cycle position and cell cycle progression, it is not surprising that some association between apoptosis and radiosensitivity has been observed (Lonati et al., 2021; Pawlik and Keyomarsi, 2004). However, our analysis showed a decrease of pro-apoptotic gene *BAX* mRNA expression with increasing radiation dose, indicating an absence of apoptotic response.

Despite the evidence of harmful effects of ionizing radiation on biological systems, our results showed that the radiation doses tested did not affect the progression of the oocyte nuclear maturation obtained from antral follicle. Besides, IR seems to affect the oocyte and the cells of cumulus in a different way. While the oocyte matures and apparently has not increase in DNA lesion, the two repair pathways may be activated in the cumulus cells. The mRNA abundance of some genes increases and others decrease, indicating that cells regulate these genes differently. These results are potentially important to understand the molecular basis for cell line-dependent differences in radiation sensitivity. Therefore, it is necessary to evaluate the effects of IR on pre-antral follicles, as well as the following development stages of the oocyte (fertilization, embryonic development).

## Conclusion

The ionizing radiation doses of 0.9 Gy, 1.8 Gy, 3.6 Gy or 18.6 Gy tested in this study had no apparent effect on oocyte nuclear maturation and DNA damage; nonetheless molecular pathways involved in DNA repair and apoptosis were affected by IR exposure in cumulus cells.
